# Some Remarks in Reference to the Necessity of Early and Free Venesection in the Treatment of Meningitis—with Cases

**Published:** 1856-03

**Authors:** J. W. McKinney

**Affiliations:** New Albany, Ill.


ARTICLE III.—*Some Remarks in Reference to the Necessity of Early and Free Venesection in the Treatment of Meningitis—with Cases.* By J. W. McKinney, M.D., New Albany, Ill., Jan. 1856.

In all diseases of a violent and dangerous tendency, the treatment of which is held in discrepancy, it should be the imperative duty of every member of the profession, of any considerable experience, to give, at least, the result attending his practice in any given number of cases. Such a course would assist in establishing a more uniform practice in treating such cases.

Meningitis being a formidable malady, and one by no means of rare occurrence, it is hoped the following cases, with the remarks, may not prove wholly devoid of interest.

The class of cases as here spoken of, are not of the intermittent type, or that form of the disease simulating congestive fever; or as treated of so ably under the title of "cerebro arachnitis," by Dr. Jas. Smick, in a former volume of the *North-Western Medical and Surgical Journal,* as having "its regular exacerbations and remissions, very much like bilious remittent fever. Cases presenting regular exacerbations and remissions, being by far the more numerous, and evidently depending on a miasmatic origin, I have always classed with, and treated, as a malignant form of congestive fever; and, therefore, as being amenable to the great anti-periodic—quinia. But the disease, as here spoken of, is of that acute continued character, which is generally preceded by various preliminary symptoms; as vague uneasiness, depression of spirits, tinuitus aurium, vertigo, &c.; and where the pain in the head, for the most part, exists for a shorter or longer period *antecedently to the febrile phenomena.*

As this pain becomes more intense, we find nausea and sometimes vomiting—an excited or wild expression—eyes suffused—a buzzing noise in the ears—very painful sensitiveness to sound and light, particularly the latter,—the patient closing his eyes forcibly, while the pupils, when seen, are observed to be very small, as though he was narcotized by opium. As the fever now begins to rise, which is first evinced by preternatural heat of the head and face, we sometimes have chilly rigors, (not a chill,) while the surface is covered with horrida cutis—the radial pulse is sharp, at the same time, the temporal arteries are full, during which spasmodic jerking of the eyes, muscles of the face and arms are observed. This goose-skin appearance sooner or later gives way to a uniform heat of the surface, when the spasms, which before were only light, now become increased, and in some cases very violent. Delirium sometimes precedes the spasms, as does the double vision; but more generally this accompanies or soon follows the first violent spasm.

If the disease be not intercepted, the delirium sooner or later yields gradually to a state of drowsiness, from which the patient may be aroused, so as to give vague and imperfect answers, but which finally passes into a deep state of coma, from which nothing can arouse him. The pupils, which before were small and contracted, now become dilated. Convulsions which had occurred frequent and violently now wholly cease, or are much lighter than in the early stage. Grinding of the teeth, with frequent deep sighing, are here prominent symptoms. These are accompanied by signs of great cerebral oppression and a general exhaustion—the pulse becoming frequent and thready—surface bathed in cold sweat, in which state the patient dies.

Among the first symptoms that attract the attention of the patient, are the pain in the head and constant nausea. These, together with the spasms, were the prominent symptoms in all the cases I class under the above title. The location of the pain was almost invariably referred, in the commencement, to the *coronal*, *temporal*, or *frontal* region, and *not* to the base or back part of the head, as is the case when of an intermittent or remittent form, and evidently of miasmatic origin.

Having attended 29 well defined cases of meningial inflammation, as above described, in a practice of eight years, I give, in the subjoined table, the cases as they occurred each year. This may serve, at least, to show at what particular season the disease is most likely to prevail, and with what mortality.

NO. OF CASES OCCURRING EACH MONTH.

| Years. | Ja. | F. | M. | A. | M. | J. | J. | A. | S. | O. | N. | D. | Died. | Recov'd. | Total. |
|---|---|---|---|---|---|---|---|---|---|---|---|---|---|---|---|
| In 1848, | | | | | | | | | | | 1 | 1 | 2 | | 2 |
| In 1849, | 1 | | 2 | | | | | | | | | | 1 | 2 | 3 |
| In 1850, | 1 | | | | | | | | | | | 2 | 1 | 2 | 3 |
| In 1851, | 2 | 2 | 1 | | | | | | | | | 1 | 1 | 5 | 6 |
| In 1852, | 2 | 3 | | 1 | | | | | | 1 | | 1 | 2 | 6 | 8 |
| In 1853, | 1 | | 1 | | | | | | | | | | | 2 | 2 |
| In 1854, | | | | 1 | | | | | 1 | | | | 1 | 1 | 2 |
| In 1855, | | | | | | | | 1 | | | 2 | | 1 | 2 | 3 |
| Total. | 7 | 5 | 4 | 2 | | | | 1 | 1 | 1 | 3 | 5 | 9 | 20 | 29 |

It may be well to state, that in the above enumerated cases, the greater number were children under twelve years old. There were eight under five years, and ten between five and twelve years old. Of these, eight or nearly one-half died; while of the remaining eleven cases, which were all between fifteen and forty years old, but one died.

The difference in the relative mortality of children, and those of adolescence and adult age, it will be seen, is very great. This difference in part is undoubtedly owing to the greater susceptibility of children, and the naturally increased tendency of blood to the brain and its membranes during the period of childhood. But I attribute a portion of this greater mortality to the fact of the patient not having had the benefit of the antiphlogistic treatment, by venesection, at an earlier period after the attack. With this exalted susceptibility, and increased encephalic and meningial circulation, we could but expect this alarming and dangerous form of inflammation to be the more early established, and if not promptly relieved by depletion, that this class of patients would the sooner pass irretrievably beyond the control of our art. In all these cases, with a very few exceptions, it was twelve, twenty-four, and in some even forty-eight hours from the time the symptoms became violent, before I saw the patients. Of the cases occurring in children, the symptoms indicating effusion into the brain were present in seven, at the time of my first visit. I did not, therefore, practice phlebotomy in any of this number. The treatment adopted under these circumstances was, to prescribe an efficient dose of hydrarg. sub. mur. where the bowels had not been freely evacuated recently by other means. At the same time, to apply emplas. epis. to the temples, with ice or snow to the front and top of the head; and where neither of these could be conveniently obtained, I ordered cloths diped in cold water to be applied. After the bowels had been freely operated, small portions (from 1 to 3 grs.) of calomel were ordered every 4 to 6 hours until 7 or 8 doses were given. Where this kept up too much action of the bowels, it was discontinued earlier, and a solution of iodide potassa, in the proportion of about 20 grs. to the ounce of water prescribed, in dessert spoonful doses, every five or six hours. Quinine, whenever it could be judiciously used in large or small doses, was promptly prescribed. This, with the use of oil turpentine, applied along the whole course of the spine, together with sinapisms to the extremities, constituted the principal treatment for these cases. Suffice it to say, that all this number (seven) died except one.

There were four of the remaining cases in whom I did not practice venesection. One of these died, and three got well. Thus, it is shown, that in eleven cases where bleeding *was not* practiced, seven died. While in the eighteen cases, where free venesection *was* practiced, but two died.

As a striking example of the efficacy of venesection, to arrest meningial inflammation, I give the following case in detail:

Was called, Nov. 13, 1855, to see Mr. J. F., aged 39 years, farmer, of a plethoric constitution, with an increased corpulency beyond that of an ordinary fleshy person, who had been attacked on the evening of the second day previous, with headache, nausea, and general lassitude—what he supposed to be "sick headache." During the day and night previous to my visit, all these symptoms became gradually but greatly increased, with occasionally chilly rigors and vomiting.

The following were the symptoms as presented on the morning of the 13th:

Frequent small pulse, lancinating pain in the head, eyes suffused with tears, with small contracted pupil and total inability to behold any object in the room; very sensitive to the slightest noise, with tinnitus aurium, or rather a crashing sound in the ears; surface of the whole body and extremities, preternaturally cold, and presenting a horrida cutis appearance; tongue loaded with a white coating; constant nausea, with frequent vomiturition.

Occasionally there was light clonic spasms of the muscles of the superior extremities, lasting from a half to one minute, during which the eyes were drawn in their orbits, spasmodically to the left side.

In consequence of the state of the pulse, and general coldness of the surface above described, I did not proceed at once to take blood from the arm, but administered an emeto-cathartic of ipecac, 20 grs., calomel, 60 grs., forthwith, and applied sinapisms to the wrists and ankles, ordering dry friction with flannel cloths, to be made on the extremities, and cloths diped in cold water, to the head. After emesis was produced once or twice, ordered mustard over the stomach, and as soon as vomiting ceased, a tablespoonful and a half of oil with 30 gtts. spts. turpentine, to be administered. I now left the patient, giving orders that so soon as the pulse became full, to send a messenger and I would again visit. At nine o'clock the following night, the messenger came, requesting me to go in great haste, stating "that Mr. J. F. had fits." When I arrived, I found him in a state similar to that just succeeding a violent epileptic convulsion. On enquiry of one of the friends present, I learned he had had no less than four or five spasms within the last hour and a half. The medicine given in the morning operated kindly, producing emesis three times, and evacuating the bowels freely four times. Finding my patient now with a full, bounding pulse, and during the intermission between spasms, requiring the effort of three men to keep him on the bed, and from doing violence, I prepared, as speedily as possible, to bleed him. His head being elevated—cloths wet with cold water constantly applied thereto, and mustard drafts to the extremities; all were not sufficient to protect him against another spasm, in which every muscle of the whole frame was most violently convulsed. So soon as the relaxation of the muscles would permit my doing so, I corded the arm and opened a large orifice in the median cephalic vein, from which I drew away three and a half pints (56 oz.,) of blood. Some pallor of the lips and face being observable, and the pulse considerably softened, I closed the orifice, and was proceeding to bind up the arm, when another spasm came on. I immediately tore away the bandage, and suffered the blood to flow again till two pint cups (32 oz.,) were filled. This produced a decided impression, and at once put an end to all further convulsions. The bed being so arranged as to keep his head and chest still elevated, I ordered a continuation of the refrigerant application of water as long as the temperature should remain morbid, with the renewal of the drafts to the extremities.

Nov. 14th, 8 o'clock, A. M. Patient has remained tolerably quiet during the latter part of the night; had swallowed nothing since last evening. Pulse frequent and quick, but easily compressed; heat of the surface a little above that of the natural temperature; delirious and more restless than during the after part of the night. Ordered cold water and drafts to be continued as before, with oil of turpentine along the whole course of the spine, and as soon as patient could be got to swallow, to give 20 gr. calomel in a tablespoonful of oil.

4 o'clock, P. M. Had revived slightly, so as to take a spoonful of water and the greater portion of the dose left in the morning. I now ordered repeated doses of oil every three hours, till the bowels were operated, and then 6 grs. iod. potas. in two ounces of water, every four hours.

15th. Patient better. Oil and calomel had operated twice; had taken five doses of the iod. solution; slight mercurial feter on the breath; drowsy. When aroused, answers all questions, in a slow undertone, by "No."

Continue use of iod. solut., and occasionally refrigerant to the head.

16th. Better. Answers questions more rationally; complains some of sore mouth; says that any noise in the room produces a confused, unpleasant sensation in his head.

Continue treatment; only prolonging the time of giving the solut. to every six hours.

17th. Decidedly better. Asks for something to eat; permitted him to take some rice gruel. Directed iodide to be continued in diminished doses; also, three or four gr. doses of quinia, twice daily, as a preventative against the deleterious influence of malaria.

From this time on, he rapidly gained the equllibrium of health.

The amount of blood taken from this patient was vastly greater than I had ever before taken at one bleeding, under any circumstance. This though, will not be very surprising, when it is borne in mind that the individual was a very large, fleshy man, weighing over two hundred and forty pounds, and of a strong sanguine temperament.

Now, in this case, which in point of violence, was a little above an average, the force of the inflammation was at once cut short, if not entirely put an end to, by the free bleeding; as indeed it was in nearly all the cases where venesection was practiced. Of course, I do not mean to say that the other means used were wholly useless; but on the contrary, that they were essentially available auxiliaries. Yet, I cannot comprehend how those secondary means, (calomel, iod. potas., quinine, cold water, &c.,) would of themselves cured my patient of this inflammation, aside from general depletion; seeing that seven out of eleven died where this had been entirely omitted; although these patients had the full benefit of those other reputed means of cure, as best my judgment and experience, aided by the written authorities, could dictate.

The great necessity of an early and free bleeding, as here set forth, is only corroborative of our best medical authorities, of the present day. Dr. Dunglison, in speaking of the treatment of inflammation of the brain and its membranes, says: "As in other cases of severe internal inflammation, bloodletting is the sheet-anchor of the practitioner; and it must be pushed so as to produce a decided effect; the effect, rather than the amount of blood taken, being the guide." Dr. Wood, in his practice of medicine, says: "It is of the utmost importance to begin the treatment early. By energetic means, at this period, the patient can generally be saved. Beyond all comparison, the most effectual remedy is bleeding." And again, in still speaking of the treatment of meningitis, this same author says: "I would repeat, that a vast deal depends, as to the result of treatment in acute meningitis, upon early and vigorous bleeding, both general and local."

A number of others, equally reliable for their sound medical teaching, might be quoted, who recommend free venesection in the treatment of this disease as being the principal. But as this article has already grown far beyond what I had intended, I desist, by simply stating: that in meningitis, presenting the above symptoms, to bleed, bleed! Bleed early and freely, and stop this inflammation; and then use "other reputed means of cure" afterwards.

